# Medical Jurisprudence in America in the Nineteenth Century*Contributed to the Thirteenth International Medical Congress of Paris, 1900 and read before the Section on Legal Medicine of the Congress.

**Published:** 1900-11

**Authors:** Clark Bell

**Affiliations:** President of the International Medico-Legal Congress of New York, June, 1899; President of the International Congress of Medical Jurisprudence, at Chicago, 1893; President of the Congress of Medical Jurisprudence of September, 1895, at New York; President Medico-Legal Society of New York; Delegate from the Government of the United States to the Thirteenth International Medical Congress of 1900, at Paris; Honorary Member of the Medico-Legal Society of France, etc., etc.


### Abstracts and Selections.

### Medical Jurisprudence in America in the Nineteenth Century.*

BY CLARK BELL, ESQ., LL. D.,

President of the International Medico-Legal Congress of New York, June, 1899; President of the International Congress of Medical Jurisprudence, at Chicago, 1893; President of the Congress of Medical Jurisprudence of September, 1895, at New York; President Medico-Legal Society of New York; Delegate from the Government of the United States to the Thirteenth International Medical Congress of 1900, at Paris; Honorary Member of the Medico-Legal Society of France, etc., etc.

Medical jurisprudence is more greatly indebted to the Italian scholars of the 16th century, and the preceding centuries, than to those of any other land.

That splendid diplomat, scholar, statesman, musician and physician, Paul Zacchias, was the physician to the Pope in 1620. His treatise, now in the library of the Medico-Legal Society in New York, could be read with profound interest, to see how far advanced was this learned and gifted man, in the elementary and fundamental truths of the science, as recognized today.

From the 12th to the 16th centuries, Italy stood pre-eminent in every field of scientific study, and especially in medicine.

Prior to 1500 there were sixteen universities in Italy; while in France there were but six; in Germany, eight; in Great Britian, only two; so that Italy had then as many as England, France and Germany combined, and the supremacy of the Italian universities was above all comparison with the others.

---

*Contributed to the Thirteenth International Medical Congress of Paris, 1900 and read before the Section on Legal Medicine of the Congress.

This Italian superiority continued until after the 16th century, and it existed when Zacchias wrote and flourished.

It has been well said that "medical jurisprudence owes its power to knowledge derived from every branch of medicine; but the law determines how far this power shall be utilized in the administration of justice."

The superiority attained by Italy, that made her the home of science and arts, in the 14th, 15th, 16th and 17th centuries, was due to the more favorable laws, and the liberal and fostering influence of the Popes of Rome in these eras.

Germany felt this influence, and when, in 1533, the "Constitutio Criminalis," of Charles V, of 1532, was first published. To the present day, the German Fatherland owes its pre-eminence in medical jurisprudence to favoring legislation, and the friendly fostering care of the governments. The "Constitutio Criminalis" of Charles V made it obligatory on the courts to take the evidence of medical men in medico-legal cases.

For more than two centuries Germany has had an organization of medico-legal officials who are required by law to be qualified by an especial education, not only to procure the medical facts needed by the courts, but to estimate their weight for the benefit of the courts.

In 1650 Machiavelli delivered the first course of lectures on legal medicine at the University of Leipzic, and he was succeeded by Bohn; and as early as 1720 professorships of forensic medicine were erected by the government of Germany.

The literature of the science was enriched, in the 18th century (1725), by the celebrated authors of Valentini, Teichmeyer and Albertus, one hundred years later than the writings of Zacchias. These writers laid the foundation of that literature of forensic medicine for the German scholars, exceeding that of nearly every country, and which, in later years, in the 19th century, has been quickened by the clinical schools, the first of which was established at Vienna, at about 1830; at Berlin, in 1833; at Munich, in 1865. Germany owes her great advancement to the fostering hand of the government, and to favoring legislation.

France, from 1570 to 1692, also enacted laws which favored the study, culture and advancement of legal medicine, and the science progressed; but at the close of the 17th century, the medico-legal officials became hereditary, and the science languished until after the French Revolution.

Since 1790, however, no nation on the globe has surpassed or even equaled France, in the culture and advancement of medical science and the arts; and from the era of the French Revolution, and under the reign of the great Napoleon, and those who have succeeded him, France advanced, until she became the seat and centre of the highest civilization in the nineteenth century, which proud position she has continued to occupy.

The Exposition of 1889, and the splendid one which marks the close of the 19th century, well illustrate her place in the world of science and arts, and the advancing civilization of the world, as the 20th century opens its portal to the brotherhood of man in all countries upon the globe.

During all these years of advancing supremacy in France, all was due to the splendid support given by the government, and by the generous laws of France, and forensic medicine has kept pace with the sister sciences there.

Chasseur, in 1790, was the first lecturer in legal medicine, and Mahon, in 1795, was the first professor of medical jurisprudence.

In Great Britain very little attention was paid to medical jurisprudence, in the 18th century, and she transmitted to her American colonies laws that have been well stated by Prof. Stanford E. Chaille, in his centennial address before the International Medical Congress of September, 1876, intended as a review of the preceding one hundred years of forensic medicine, as: "barbarously conspicuous for the absence of provisions to apply medical knowledge to the administration of justice, and Anglo-American law continues to be, in large measure, hostile to medical jurisprudence."

These words were spoken in 1876—twenty-five years ago. They properly describe the state of the law of Great Britain at that date, and show why, in the American States the 19th century opened in America with the standard of forensic medicine trailing in the dust. When contrasted with France, Germany and Italy, but America, with this sad inheritance from the mother country, has not remained where she was placed in the first fifty years of the American national life.

Scotland should be exempt from these criticisms. In 1803 a chair of forensic medicine was founded in the University of Edinburgh. Dr. Duncan, the elder, was the first lecturer on medical jurisprudence, in 1801; and his son the first professor, in 1802.

While this University was chartered in 1682, it was not until 1726 that an English medical faculty was established, with lawful authority to confer degrees. Before 1726, and after 1705, a few honorary degrees had been conferred, but not by legal authority.

In each of the twenty-three medical colleges existing in England, Ireland and Scotland, in 1875, there was a regular teacher devoted to forensic medicine, on whom legal authority to confer a degree in State medicine was conferred upon those at Cambridge, Oxford, Edinburgh and Dublin; but it cannot be said that medical jurisprudence, as a science, was practically taught outside of the University of Edinburgh, if there, except as to toxicology.

Scotland has kept alive, on her altar at Edinburgh, the science, and Prof. Ogston, and the Scotch scientists, have been among the foremost of medico-legal jurists of Great Britain in the earlier years of the century.

The Registration Act was not passed in England until in 1858, and "Glenn's Manual of the Laws Affecting Medical Men," London, 1721, cited by Professor Chaille, in his admirable address, from which I quote, freely states and details thirty-six laws, from 1850 to 1870, of which twenty-five were passed in the twenty years between 1850 to 1870. The medical profession never had legal recognition under English laws until 1858. No laws existed for the preparation of official medical experts.

The British bench never held medical men or medical expert evidence with the respect with which it was received in France, Germany and Italy, under the provisions of law; and there has been no legislation in England tending to utilize the aid of competent medical knowledge, in aid of the administration of justice, in the British Islands, save in Scotland.

At the opening of the 19th century, the science of medical jurisprudence, upon the American Continent, did not command that recognition in either profession of law or medicine that its importance demanded.

At this era, the science may well be said to be at its decadence in all the English speaking peoples of the world. In England, the works of Farr, Dease, Male and Haslem may well be said to reflect the progress and state of the science, and the American States cannot be said to have made any substantial advances in forensic medicine, having but recently emerged from that revolutionary struggle which established the American nation upon the principles of human rights enunciated in the Declaration of American Independence.

Dr. Benjamin Rush, of Philadelphia, one of the signers of that Declaration, was one of the most conspicuous of those who gave the science attention. He was Professor of Chemistry in the Philadelphia College of Medicine and the University of Pennsylvania, and his labors extended from 1769 until his death in 1813. He held the chairs of Theory and Practice of Medicine, and of the Practice of Physics. He left sixteen lectures, the last of which was delivered in 1810 at the University of Medicine, and was devoted to the science of medical jurisprudence. Its chief features relate to mental states, testamentary capacity and legal responsibility in cases of crimes committed.

Dr. Thomas Cooper, who had been a judge in the courts of Pennsylvania, was a Professor of Chemistry and Mineralogy in the University of Pennsylvania, and published "Tracts on Medical Jurisprudence."

In 1829, Dr. J. Bell, also of Philadelphia, published an address upon the same topic, and he delivered a course of lectures on medical jurisprudence in the Philadelphia Medical Institute, of which he published a syllabus.

Professor Robert Eglesfield Griffith, M. D., of the University of Maryland, and Lecturer in the University of Virginia in 1832, edited with notes and additions the first American edition of the work of Michael Ryan on Medical Jurisprudence, which was published by Carey & Lea, at Philadelphia, where Professor Griffith then resided, and where he died in 1850, at the close of the first half of the century.

But there is no American name to whom the science of medical jurisprudence is more indebted than that of Prof. Theodore Romeyn Beck, M. D., LL. D. He was born at Schenactady, N. Y., August

11, 1791, and he died November 19, 1855. He graduated at Union College at the age of sixteen years and took his degree in medicine at the New York College of Physicians and Surgeons in 1811. Mercersburg College, Pennsylvania, and Rutgers College of New Jersey conferred the degree of LL. D. upon him.

In 1815 he was made lecturer on medical jurisprudence in the College of Physicians and Surgeons of New York, and in 1826 he was made Professor of Medical Jurisprudence in that college, and held that chair until 1840.

In 1823 he published "Beck's Medical Jurisprudence," which passed through many American, English and German editions, and which was universally acknowledged, in its day, to be the leading work on the science, and was found as often upon the shelves of the lawyer's library as those of the medical man.

For forty years of his life this illustrious man devoted himself to the advancement of the science, and most of it in the latter part of the first half of the century.

In 1853, at Albany, the capital of New York, in addressing the Legislature of his native State upon the proposed establishment of a university, he demanded "the appointment, under public authority, of a Professorship of Medical Jurisprudence or Forensic Medicine," and in support of it said: "There is a person now living (Orfila), the subject of whose knowledge on the power of poisons is such that he is not only called to examine cases in any part of France, but not long since was summoned to Belgium in one, which at the time attracted the attention of all Europe. I hold that there should be two or three persons of this character appointed and paid by the government, to perform this important duty."

In considering the century we can best understand the progress of the science by dividing it into three eras, considering the first third, as we have done, and in the labors of Professor Beck, and advancing it into the second third and a few years beyond the first half of the century.

The most illustrious name in forensic medicine, after that of Beck, in the department of the medical jurisprudence of insanity, was that of Isaac Ray, M. D. He stands at the very head of American alienists. His treatise on "The Medical Jurisprudence of Insanity" has never been surpassed, in the century in which he lived and acted, in any country, and no work of the century produced a more profound or lasting impression upon the mind of men.

He lectured on insanity at the Jefferson Medical College, Philadelphia, and his contributions to *Mental Pathology*, published in 1873, enriched the literature of this branch of forensic medicine.

In considering medical jurisprudence from this standpoint, we should classify it into general divisions, and as one of these, "The Medical Jurisprudence of Insanity," Dr. Ray stands pre-eminent.

With his name should be mentioned some of the earlier alienists: Pliny Earle; Allan McLane Hamilton; Henry P. Stearns; Dr. John P. Gray, of Utica, N. Y.; Prof. Chas. H. Hughes; William A. Hammond; Prof. Charles K. Mills, of Philadelphia, Pa.; Dr. W.

W. Godding; Dr. Geo. M. Beard; Dr. Nichols, of Bloomingdale Asylum, New York City; and a great body of earnest students and workers among the superintendents of American asylums and hospitals for the insane, with many names of merit among the students of neurology and psychiatry that have adorned the brilliant galaxy of American students of mental medicine.

Perhaps no work of recent date exercised a greater influence in the nineteenth century in America than did the work of Dr. Alfred Swayne Taylor, on medical jurisprudence. The work had a greater influence than the volumes put out by John C. Bucknill and Dr. Hack Tuke, which was a work of great value and entitled to high praise. The splendid work of Chaude and Briant, which was also a contemporary, being in the French tongue, could not rival these publications.

"Taylor's Medical Jurisprudence" was universally recognized as the model standard, and superseded Beck, who had so long stood at the very front. It passed through a large number of American editions, and its 12th American edition was completed in 1897.

Among medico-legal jurists, who have been prominent, I should name in the last third of the century, Chief Justice Charles Doe, of the Supreme Court of New Hampshire, to whom (more than all other American jurists combined) we are indebted for the overthrow, in many of the American States, of the innovation made in the law of England after the McNaughten case, making the knowledge of right and wrong the test of criminal responsibility of the insane.

His associate, Judge Ladd, of the Supreme Bench of New Hampshire, and Judge H. M. Somerville, of the Supreme Bench of Alabama, who wrote the prevailing opinion of the court of that State, asserting what has come to be known as the New Hampshire doctrine, is entitled to our tribute of praise and commendation. Among the eminent men of the bar, who have taken part in the labors of the society and in advancing its work may be named: Hon. David Dudley Field; Hon. E. A. Stoughton; Judge A. L. Palmer, of the Supreme Court of New Brunswick; Sir John C. Allan, Chief Justice of that bench; Judge W. D. Hardin, of Savannah, Ga.; Judge Charles P. Daly, and Mr. Austin Abbott. Of those whose labors have ceased, not to mention the lustrous and brilliant names of those still living, and among medical men: Dr. James C. Wood; Dr. Frank H. Hamilton; Dr. Carnochan; Dr. S. W. B. McLeod, and Dr. Fordyce Barker.

Consider the views of Chief Justice Charles Doe, of the Supreme Court of New Hampshire; Associate Justice Ladd, of the Supreme Bench of that State; Judge H. M. Somerville, of the Supreme Court of Alabama; Judge Montgomery, of the Supreme Court of the District of Columbia, and Judge Dillon, of Iowa, all members of the Medico-Legal Society; Shaw, of Massachusetts; Edmonds, of New York; Bell and Perley, of New Hampshire, in contrast with the language of the Lord Chancellor of England as late as 1862, who, in referring to insanity in the English House of Lords, declared that "The introduction of medical opinions and medical theories into this subject has proceeded upon the vicious principle of considering insanity as a disease," and he condemned the "evil habit that has grown up of assuming that it was a physical disease."

And it was less than 100 years prior to this utterance (1769) Lord Chancellor Blackstone said: "To deny the possibility, nay actual existence, of witchcraft and sorcery is to at once flatly contradict the revealed word of God," and also added, "And the thing itself is a truth to which every nation in the world hath in its turn borne testimony."

The following are among the eminent French collaborators in the work of the Medico-Legal Society: Dr. T. Gallard, Ex-Secretary of the Medico-Legal Society of France; Dr. Louis Penard, Ex-President of the Medico-Legal Society of France; Dr. Devergie, Ex-President of the Medico-Legal Society of France; Mr. Ernest Chaude, Ex-President of the Medico-Legal Society of France; Dr. Devilliers, Ex-President of the Medico-Legal Society of France; Prof. Dr. Benj. Ball, author; Dr. Emile Hourteloup; Dr. Lunier; Dr. Briere de Boismont; Dr. Chevalier; Dr. Legrard du Saille; M. Hemar, Ex-President of the Medico-Legal Society of France; Dr. August Voisin; Dr. Carpenter; Prof. Dr. Luys; Marcel Briand, and Dr. Foville.

Living members of eminence identified with the progress of medical jurisprudence in America: Prof. Brouardel; Dr. Motet; Dr. Lutaud; Dr. A. Leblond; Dr. Magnan; Dr. Ritti; Dr. Christian; Dr. Bertillon, and Dr. Forel.

The following are deceased honorary members of the Medico-Legal Society: Prof. Francis Wharton, writer on international law; Dr. Devergie, President of the Medico-Legal Society of France; M. Barnier, President High Court of Cassation of France; Dr. Louis Penard, Ex-President of the Medico-Legal Society of France; Prof. Charcot, Paris; Sir James Fitz James Stephen, of London, England; Dr. Frank H. Hamilton, of New York; Hon. Charles P. Daly, of New York; Dr. John C. Bucknill, Chancellor's Visitor in Lunacy, London; Dr. D. Hack Tuke, of London, and Dr. Fordyce Barker, of New York.

### TOXICOLOGY.

The most lustrous names among the great chemists of the American people are those of Prof. Wormley, of Philadelphia; Prof. John J. Reese, of Philadelphia; Prof. R. Ogden Doremus, Ex-President of the Medico-Legal Society of New York; Prof. Victor C. Vaughan, of the University of Ann Arbor, Mich., and Dr. Henry Leffman, of Philadelphia.

Professor Theodore G. Wormley succeeded Prof. R. R. Rogers in the Chair of Toxicology in the Medical Department in the University of Pennsylvania in 1877, and he filled it with distinction until his death in 1897. His work upon "Micro-Chemistry of Poisons" is a monument of learning, and is universally regarded as a standard authority by the professions and the courts. His contribution to the

International Medical Congress, in 1876, in Philadelphia, was "Medical Chemistry and Toxicology."

Professor John J. Reese was born June 16, 1818, and he died September 4, 1892. He was the foremost medical jurist and toxicologist of his day in Philadelphia, and the author of a text-book on Medical Jurisprudence and Toxicology. He was President of the Medical Jurisprudence Society of Philadelphia; an active and honorary member of the Medico-Legal Society of New York; and he edited the seventh and eighth American editions of "Taylor's Medical Jurisprudence," and was a prolific writer on subjects connected with medical jurisprudence. He held the Chair of Medical Jurisprudence and Toxicology in the Auxiliary Department of Medicine in the University of Pennsylvania from 1865 to October, 1891.

Professor Vaughan, of the University of Ann Arbor, is one of the most brilliant of the chemists of our era. He is a member of the Medico-Legal Society of New York.

Dr. Henry Leffman, of Philadelphia, was Lecturer on Toxicology at the Jefferson Medical College from 1875 to 1883, and Chemist to the Coroner of Philadelphia from 1885 to 1897. He edited the fourth edition of Professor Reese's text-book on "Medical Jurisprudence and Toxicology."

Professor R. Ogden Doremus is the Nestor of American chemists and toxicologists. He has held the Chair of Medical Jurisprudence and Toxicology in the College of the City of New York and at Bellevue Medical College for a great many years. He was Chemist of the Medico-Legal Society for many years, and held the office of President of that body.—*From Advance Sheets of the Medico-Legal Journal.*

